# Genomic basis of seed colour in quinoa inferred from variant patterns using extreme gradient boosting

**DOI:** 10.1111/pbi.14267

**Published:** 2024-01-11

**Authors:** Felix L. Sandell, Thomas Holzweber, Nathaniel R. Street, Juliane C. Dohm, Heinz Himmelbauer

**Affiliations:** ^1^ Department of Biotechnology, Institute of Computational Biology University of Natural Resources and Life Sciences (BOKU) Vienna Austria; ^2^ Department of Plant Physiology, Umeå Plant Science Centre Umeå University Umeå Sweden; ^3^ SciLifeLab Umeå University Umeå Sweden

**Keywords:** quinoa, genome sequencing, machine learning, seed colour, betalain synthesis pathway, genotype‐phenotype relationships

## Abstract

Quinoa is an agriculturally important crop species originally domesticated in the Andes of central South America. One of its most important phenotypic traits is seed colour. Seed colour variation is determined by contrasting abundance of betalains, a class of strong antioxidant and free radicals scavenging colour pigments only found in plants of the order Caryophyllales. However, the genetic basis for these pigments in seeds remains to be identified. Here we demonstrate the application of machine learning (extreme gradient boosting) to identify genetic variants predictive of seed colour. We show that extreme gradient boosting outperforms the classical genome‐wide association approach. We provide re‐sequencing and phenotypic data for 156 South American quinoa accessions and identify candidate genes potentially controlling betalain content in quinoa seeds. Genes identified include novel cytochrome P450 genes and known members of the betalain synthesis pathway, as well as genes annotated as being involved in seed development. Our work showcases the power of modern machine learning methods to extract biologically meaningful information from large sequencing data sets.

## Introduction

Quinoa (*Chenopodium quinoa*) is a eudicot plant affiliated with the order of Caryophyllales. It is one of several agriculturally important crops of the family Amaranthaceae comprising also, for example, sugar beet, table beet, chard, spinach and amaranth. Quinoa was domesticated in the Andes (Pickersgill, 2007) and grows natively in high‐altitude regions of Bolivia, Ecuador and Peru and at sea level in Chile. Due to increasing demand for quinoa, which is considered a healthy dietary alternative to cereals (Gordillo‐Bastidas, 2016), efforts are underway to cultivate quinoa outside of South America, but as of now the worldwide demand for quinoa is supplied mainly by Peru [100.000 tons in 2021 (*Quinoa Production in Peru 2020*, n.d.)] and Bolivia [70.000 tons in 2020 (*Quinoa Production Bolivia 2020*, n.d.)].

Quinoa is a tetraploid species with 2*n* = 4*x* = 36 chromosomes and an estimated genome size of 967 Mbp (Yangquanwei *et al*., 2013). It emerged from the hybridization of two unknown diploid species of the genus *Chenopodium* 3.3–3.6 million years ago, and both subgenomes are well conserved in the hybrid (Jarvis *et al*., 2017; Schiavinato *et al*., 2021). Genomic resources available for quinoa include reference genome assemblies for the Chilean accession PI 614886 (QQ74; Jarvis *et al*., 2017) and the Bolivian accession CHEN125 (Bodrug‐Schepers *et al*., 2021) and re‐sequencing data in combination with phenotyping of 310 quinoa accessions (Patiranage *et al*., 2022).

Patiranage *et al*. (2022) provided phenotypic data of 17 important traits but did not consider one of the agriculturally most important traits in quinoa, seed colour. As in lentils, seed colour is related to texture and taste of cooked quinoa, and therefore different dishes require differently coloured quinoa. It was recently shown that seed colour is determined by the presence of two types of betalains, betaxanthins (yellow) and betacyanins (purple), in the seed coat (Escribano *et al*., 2017). Betalains are produced in species of the Caryophyllales order and, similar to anthocyanins (the pigments widely produced in other plant species) and other flavonoids and tannins, are strong antioxidants with high anti‐inflammatory and free radical scavenging activity (Gordillo‐Bastidas, 2016). Since betalains have never been found in plants that produce anthocyanins it has been concluded that betalain synthesis and anthocyanin synthesis are mutually exclusive pathways (Brockington *et al*., 2015; Stafford, 1994; Timoneda *et al*., 2019). The simultaneous presence of both types of betalain pigments leads to orange and reddish quinoa seeds, whereas white quinoa seeds lack pigments altogether (Escribano *et al*., 2017). Pre‐clinical work suggested health benefits of betalains in table beet by exhibiting chemopreventive properties in the context of cancer (Lechner and Stoner, 2019). The natural bioavailability of betalains is low and therefore they are a desired supplement in human diet. Betalains are also a sought‐after colouring agent for the food industry (Calva‐Estrada *et al*., 2022).

The first step of the betalain synthesis pathway starting from tyrosine is the hydroxylation of tyrosine to L‐DOPA catalysed by cytochrome P450 enzymes. The second step is either the formation of betalamic acid from L‐DOPA catalysed by the enzyme L‐DOPA 4,5‐dioxygenase (DODA) or, alternatively, the oxidation of L‐DOPA to cyclo‐DOPA, which is also catalysed by a cytochrome P450 enzyme. Betalamic acid can then react spontaneously with an amino or amine group to form yellow betaxanthins. There are two ways to build betacyanins from betalamic acid, both require cyclo‐DOPA: either betalamic acid reacts spontaneously with cyclo‐DOPA to betanidin followed by an enzymatic step catalysed by a glucosyltransferase resulting in betacyanin, or cyclo‐DOPA is glucosylated by a glucosyltransferase followed by spontaneous reaction with betalamic acid to betacyanin (Timoneda *et al*., 2019).

It is known that a cytochrome P450 family gene (CYP76AD1) controls betalain production in table beet (Hatlestad *et al*., 2012). It facilitates the synthesis of cyclo‐DOPA from L‐DOPA. Silencing of CYP76AD1 removes the red colour from table beets (Hatlestad *et al*., 2012) resulting in the sole expression of yellow betaxanthins. It has been shown that the quinoa gene CqCYP76AD1/AUR62012346‐RA, whose sequence is highly similar to beet CYP76AD1 and could be isolated from the hypoctyl of young quinoa plants, controls the colour of quinoa stems (Imamura *et al*., 2018; Patiranage *et al*., 2022). However, it is unclear whether CqCYP76AD1 also plays a role in betalain synthesis in seeds.

The recent rise in the possibility of re‐sequencing genomes in large numbers also sparks a need for sophisticated data analysis methods. In the past few years machine learning algorithms have been used to efficiently and successfully analyse large and complex biological datasets. It has been shown that machine learning models can play an important role in understanding the complex nature of genetic traits and can serve as a powerful alternative to traditional univariate Genome Wide Association Studies (GWAS; Novakovsky *et al*., 2023; Shen *et al*., 2022). When used as a ‘black box’ approach machine learning can provide highly accurate predictive models from large‐scale, noisy data and where complex interactions determine the phenotype. A number of methods now also allow determining ‘feature importance’, which removes the black box nature of machine learning and enables identification of candidate ‘features’ for downstream functional validation. As such, machine learning can be used both as a predictive method and as an approach to identify candidate causative variants. Gradient boosted trees possess the capability to discern complex interactions among features (in the context of the current study, ‘features’ are single nucleotide polymorphisms or SNPs) and have demonstrated particular efficacy in the analysis of multi‐locus traits and do not necessitate assumptions about hereditary components (Grinberg *et al*., 2020).

Our results provide insights into the genetic basis of seed colour in quinoa by application of the gradient boosting machine learning algorithm XGBoost (Chen and Guestrin, 2016; Friedman, 2001) on genomic variants discovered in 156 re‐sequenced quinoa accessions. Here, we report a novel candidate cytochrome P450 gene cluster in quinoa involved in betalain synthesis and identify a number of additional candidate genes associated with variation of betalain levels in quinoa seeds. These genes could be valuable targets for further efforts in quinoa breeding. Furthermore, to our knowledge, this is the first large genetic study in Caryophyllales that examines the genetic differences between a wide variety of accessions with differently coloured seeds.

## Results

### Re‐sequencing of quinoa accessions

We sequenced 106 quinoa accessions that originated from Bolivia and Peru and combined them with 50 previously sequenced accessions (Bodrug‐Schepers *et al*., 2021). The sequencing coverage of the 156 accessions ranged from 3.5‐fold (20 050 178 reads with a read length of 125 bp) to 7.0‐fold (40 770 980 reads) with a mean coverage of 5.2‐fold. The accessions were chosen in order to maximize diversity by using different sampling locations and by seed colour according to the available passport information of the IPK genebank. After sequencing we performed quality control and quality trimming of the sequencing data, and mapped all 156 accessions against the assembled genome of the Bolivian reference genotype CHEN125 (Bodrug‐Schepers *et al*., 2021), a white‐seeded cultivar here referred to as ‘RefCHEN125’, and performed variant calling. Subsequently, we performed combined genotyping of all accessions, filtered for minor allele frequency and sequencing coverage and created a genotype matrix.

After filtering a total of 3 943 156 variants were called on RefCHEN125 averaging to one variant position per 245 bp (four variants per kilobasepair). The amount of variation per accession varied from 470 342 homozygous variant positions in the Peruvian accession D12373 to 1 193 892 homozygous variants in the Peruvian accession D12219. On average, each accession had 842 731 homozygous variants (Table S1).

### Gene annotation and functional annotation for the Bolivian reference genotype

We established a gene set for the Bolivian reference genome RefCHEN125 using the AUGUSTUS pipeline (Stanke *et al*., 2008) with supporting information provided by expression data from Illumina mRNA‐seq and Pacific Biosciences IsoSeq data. Of the initial 52 599 predicted genes 45 439 were supported by transcript evidence. We performed functional annotation of all predicted genes based on orthology inference using a database comprising 4.4 million ortholog groups based on 5090 organisms, resulting in functional annotations for 45 636 genes of which 26 005 had Gene Ontology (GO)‐terms assigned. The combination of genes supported by transcript evidence and functionally annotated genes formed the new gene set named ‘gsCHEN125’, containing 50 163 genes with a mean genomic length of 5736 bp. The mean transcript length was 1494 bp, and the mean length of the coding sequences (CDS) was 1198 bp. On average, a transcript consisted of 5.98 exons, and 6934 single‐exon genes were identified (Table 1).

**Table 1:** Metrics for the gene prediction

Predicted genes	52 599
Predicted transcripts	58 149
Predicted genes with mRNA‐seq support or functional annotation	50 163
Predicted genes with mRNA‐seq support	45 439
Predicted genes with functional annotation	45 636
Predicted transcripts with support or functional annotation	55 627
Mean length of genes, incl. introns (bp)	5736
Mean CDS length (bp)	1198
Exons per gene	5.98
Single exon genes	6934

Genes with mRNA‐seq support were supported by expression evidence (Illumina and/or PacBio). Except for the two first lines, all numbers refer to the final gene set (comprising 50 163 genes).

According to BUSCO analysis (Manni *et al*., 2021; Simão *et al*., 2015) our gene set was highly complete with 99.6% (254 of 255) detected core eukaryotic genes and 94.8% (2206 of 2326) core eudicots genes (Table 2). We compared our gene set to the previously published gene annotation (Jarvis *et al*., 2017) for the Chilean quinoa cultivar QQ74 (here referred to as ‘gsQQ74’) consisting of 44 776 genes with a mean length of 4798 bp and a mean CDS length of 1274 bp, which was similar to the gsCHEN125 results. The BUSCO scores for gsQQ74 were slightly lower than for gsCHEN125, exhibiting 97.3% completeness for the eukaryote dataset and 92.8% completeness for the eudicot dataset. This indicates that the gene set for the Bolivian reference genome RefCHEN125 is the most complete quinoa gene set available as of now (Table 2). Finally, we compared the coding sequences of both gene sets using BLAST (Camacho *et al*., 2009) and found roughly half of the genes with identical gene structure in each genome (with 90% identity and a length overlap of at least 80%). The other half of the genes showed differences including missing exons, large genes incorrectly predicted as multiple smaller ones, or multiple small genes incorrectly predicted as one large gene. With our search settings only 17 gsQQ74 genes (three with functional annotation) and 80 gsCHEN125 genes (17 with functional annotation) were missing in the other gene set, respectively, meaning that not a single BLAST hit could be found in the set of coding sequences. We randomly selected a few of those genes and compared their sequences against the respective reference genome where they had not been predicted, and in all cases we could identify the genomic sequence of the missing gene. This indicates that these cases can be attributed to differences in the gene prediction pipelines. Another comparison was performed to a gene set of the Bolivian quinoa cultivar ‘Real’ (Zou *et al*., 2017) consisting of 54 438 genes. As this gene set was not publicly available, we compared BUSCO results based on the BUSCO dataset ‘Embryophyta odb9’. Results for both gene sets were similar, with gsCHEN125 having slightly (6) fewer complete BUSCO genes but also slightly (12) fewer missing BUSCO genes.

**Table 2:** Comparison of BUSCO results of genesets ‘gsCHEN125’ and ‘gsQQ74’ with BUSCO database eukaryota_odb10 in columns 2 and 3 and database eudicots_odb10 in columns 4 and 5

Database	eukaryota_odb10 (255 genes)		eudicots_odb10 (2326 genes)	
Geneset	gsCHEN125	gsQQ74	gsCHEN125	gsQQ74
Complete BUSCOs	254 (99.6%)	248 (97.3%)	2206 (94.8%)	2157 (92.8%)
Complete and single‐copy BUSCOs	38 (14.9%)	64 (25.1%)	489 (21.0%)	606 (26.1%)
Complete and duplicated BUSCOs	216 (84.7%)	184 (72.2%)	1717 (73.8%)	1551 (66.7%)
Fragmented BUSCOs	1 (0.4%)	6 (2.4%)	27 (1.2%)	64 (2.8%)
Missing BUSCOs	0 (0%)	1 (0.3%)	93 (4.0%)	105 (4.4%)

### Phenotyping of seed colours

**Figure 1:**
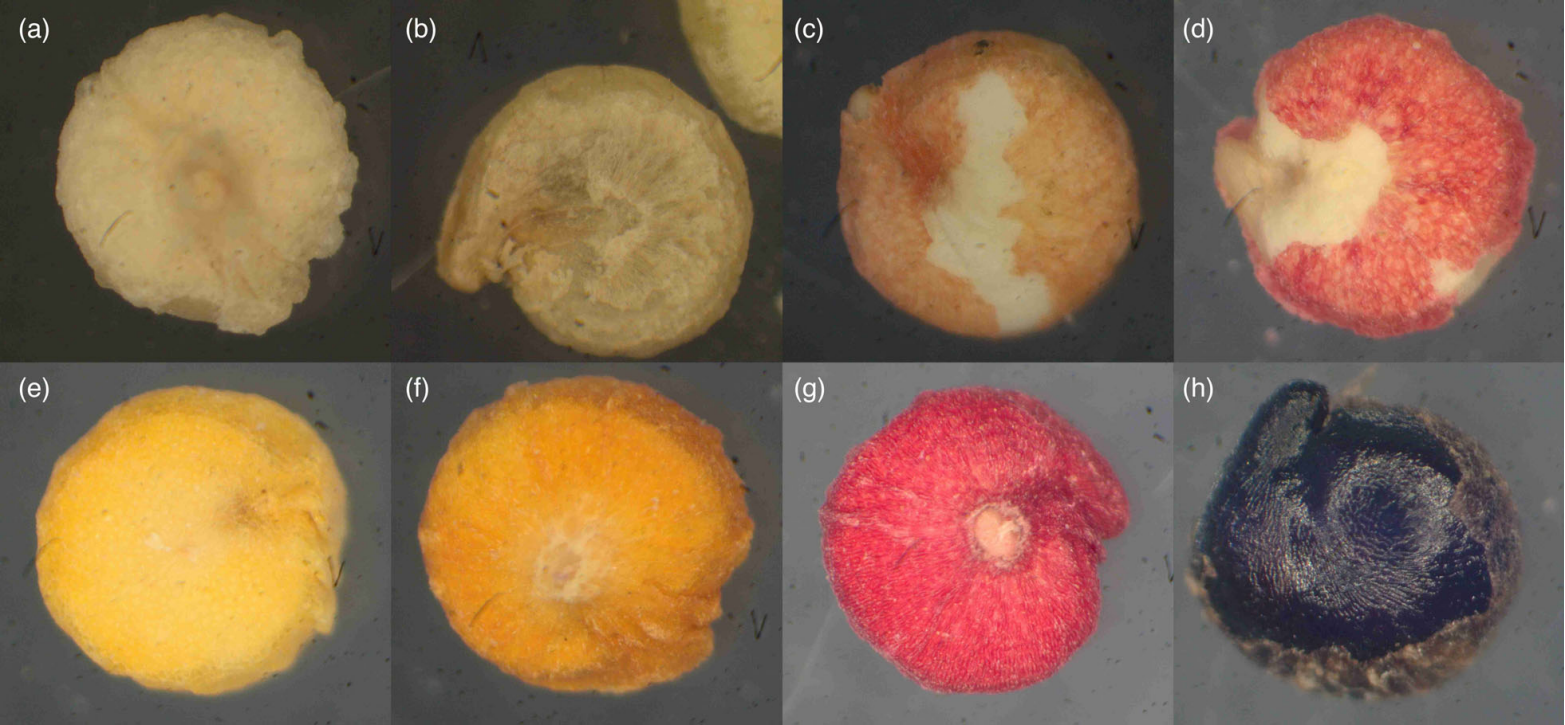
Representative quinoa seeds classified as white (a), beige (b), greenish‐red (c, d), yellow (e), orange (f), red (g) and black (h) at 50‐fold magnification.

Each of the 156 accessions analysed in this study had initial seed colour information in their passport data of the IPK genebank. We revised these 13 different colour descriptions by visual inspection of the seed samples (about 20–50 seeds per accession) and re‐classified them into nine different seed colour groups (Table 3; Figure 1).

**Table 3:** Number of sequenced quinoa accessions by seed colour

Seed colour	Number of accessions
**White**	**45**
**Beige**	**27**
Yellow	26
**Orange**	**23**
Mixed colours	9
Black	7
Red	7
Brown	6
Greenish‐red	6

The groups used for classification are printed in bold.

While the white, beige and orange seeds (Figure 1a,b,f) were quite uniform in colour, the yellow‐seeded accessions showed a wide range of yellowish colours, with some accessions being bright yellow and closer to the orange accessions, some being slightly greenish, but most of the accessions being moderately yellow and closer to the white accessions. We only had a low quantity of accessions with brown, greenish‐red, red and black seeds available (Figure 1c,d,g,h). In general, this led to an imbalanced dataset, meaning that not all groups were equally represented. The group named ‘greenish‐red’ comprised accessions that showed similar structure and size of their seeds and a mixture of red and green colour shades within each sample. Finally, there was one group of accessions where the seeds showed a variety of different colours, which we refer to as ‘mixed colours’ (Table 3).

We selected the three groups beige, orange and white for our initial analysis since they were clearly distinguishable, and all three groups had a considerable sample size. Together with the genomic variation patterns per accession, this dataset was used to infer combinations of SNPs predictive of seed colour in quinoa.

### Identifying variation patterns in quinoa

The 95 accessions that showed seed colours classified as beige (27), orange (23) and white (45) had in total 3.9 million variant positions on RefCHEN125 of which 233 were shared between all these accessions. To understand the underlying nature of the trait that could be caused by a complex combination of genetic variation we decided to use a white‐box machine learning model to determine whether seed colour can be predicted from genotype information in quinoa, and consequently learn from our models which genomic variants are of high predictive importance and therefore are potentially causative for the phenotypic differences. We used the extreme gradient boosting algorithm XGBoost (Chen and Guestrin, 2016) to predict seed colour based on SNP patterns within RefCHEN125. We expected this to be challenging since a lot of variants describing a comparatively low number of accessions (high feature‐to‐sample ratio) and working with groups of different sizes (imbalanced dataset) are known factors that impede successful model generation. Our strategy was based on the expectation that presumably several genes are directly involved in the betalain synthesis pathway because at least three enzymatic reactions are necessary to synthesize red betacyanin from tyrosine and at least two enzyme‐mediated reactions are necessary to synthesize the yellow betaxanthin (Hatlestad *et al*., 2012). We expected that additional genes would participate in controlling their expression.

In the first step, we trained a model on the three most consistent colour groups, that is, on quinoa plants with either orange, beige, or white seeds, respectively, to reduce variation and to simplify the identification of variation patterns that affect seed colour. For each group, 80% of the accessions were used as a training set, that is, comprising 20 beige‐seeded accessions, 17 orange‐seeded accessions and 34 accessions with white seeds. The remaining accessions were used as a test set, and hyper‐parameter tuning was performed using subsets of the training set (fourfold cross‐validation, see ‘Methods’ section). We increased the weights for the two under‐represented groups to minimize the bias that might have emerged due to the unbalanced dataset. The final model showed a cross‐validation Area Under the Curve of the Receiver Operating Characteristic (AUC ROC) of 0.82 with a prediction accuracy of 88% and an f1 score of 86% (Table 4) on the test set.

**Table 4:** Prediction accuracies for the three colour (beige, orange, white) classification problem

	Precision	Recall	f1‐score	Support
Beige	1	0.57	0.73	7
Orange	0.86	1	0.92	6
White	0.85	1	0.92	11
Accuracy			0.88	24
Macro avg	0.9	0.86	0.86	24
Weighted avg	0.89	0.88	0.86	24

The column support specifies the amount of samples in the test set. The f1‐score is a measure of accuracy that is calculated from the precision and the recall of the test set.

Compared to the baseline accuracy of this unbalanced multi‐class classification problem (35.6%) our results showed a significantly higher accuracy in predicting seed colours, meaning that seed colour of 88% of the seeds in our test set could be accurately predicted. This clearly shows that our model can essentially distinguish seed colours based on the genetic variant profile of a whole‐genome sequencing data set.

We extracted the ‘feature importance’ to identify the SNPs (considered as ‘features’) of the highest importance for the performance of the model. We did this by only considering SNPs which led to an increase of prediction accuracy during decision tree calculations. This resulted in 123 variant positions in the genome (out of the total of 3.94 million SNPs) that were important for prediction accuracy and most likely played a key role in controlling quinoa seed colour.

To visualize the capabilities of our model we performed principal component analyses (Pearson, 1901; PCA) and linear discriminant analyses (Fisher, 1936; LDA), that is, applying one unsupervised and one supervised dimensionality reduction algorithm, respectively. In the first step, we applied these methods to the complete dataset of 3.95 million variants to see if any preliminary grouping could be identified within the dataset, but neither PCA nor LDA sufficiently separated the three seed colour classes (Figure S1). Therefore, we could assume that detected variants predictive of seed colour would not be artefacts arising from a particular population. To test for confounding effects of population structure we included the top 10 principal components as additional ‘features’ in the analysis but could not see any discernible impact for our models. When using the reduced set of 123 SNPs that were predictive of seed colour in quinoa according to our gradient boosting model we observed improved clustering for both PCA and LDA (Figure S2).

Since the choice of accessions to be used as training set and test set severely influences the results, we calculated 100 (this number was arbitrarily chosen) models with different randomly chosen training sets (80% of the accessions) and test sets (20% of the accessions). Not all of these models performed as well as our initial model, and the average f1 score of all models was 71%. We combined the feature importance scores and created a final set of important variants by considering only variants that were scored as important in at least nine models (this number was arbitrarily chosen), resulting in a selection of 129 SNPs (Table S2). Each of the 100 models contributed between nine and 29 SNPs to the combined variant set; there was no model contributing zero or more than 50% of its own set of important variants to the final set.

**Figure 2:**
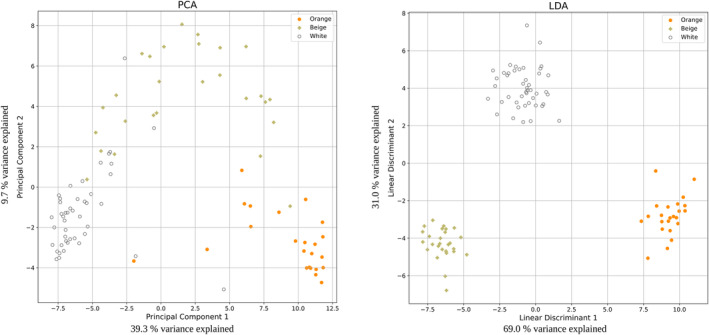
Principal component analysis (PCA) and linear discriminant analysis (LDA) of 129 SNP positions that increased the quality of at least nine independent XGBoost models using accessions with beige, orange and white seeds.

The resulting PCA and LDA plots (Figure 2) showed a notably better separation of the three groups than the initially determined 123 variants of the singular model, which shared 19 SNPs with the final variant set.

In an effort to provide further evidence supporting the high predictive capacity of this set of variants for seed colour, we conducted a retraining of all 100 models exclusively utilizing these 129 SNPs as the input feature space. This modification resulted in a notable improvement in the AUC ROC metric, which increased by approximately 16%, rising from 0.82 to 0.95. This suggests that the generated models exhibited enhanced performance after the removal of noise from the input data. The best newly trained model achieved a prediction accuracy of 0.96, a substantial improvement from the previous accuracy of 0.86. Moreover, the mean prediction accuracy across all 100 models increased from 0.71 to 0.82.

LDA, being a supervised method, outperformed the PCA and showed a perfect separation of the three seed colour groups for both feature selections of the initial model (Figure S2) and the final feature set (Figure 2). The outliers visible in the PCA were consistent with accessions that have been regularly misclassified by our models. Manual re‐inspection of the seed colour of these accessions revealed that two originally assigned to the ‘white’ group and appearing in the cluster of beige accessions in the PCA were indeed slightly darker than other white seeds and could be re‐assigned as ‘beige’.

We concluded that we had identified a meaningful set of SNPs from the re‐sequenced accessions that represented genomic variants predictive of seed colour variation in quinoa, and also that the supervised learning method gradient boosting in combination with dimensionality reduction techniques like PCA and LDA can be a viable tool to analyse complex genetic traits.

**Figure 3:**
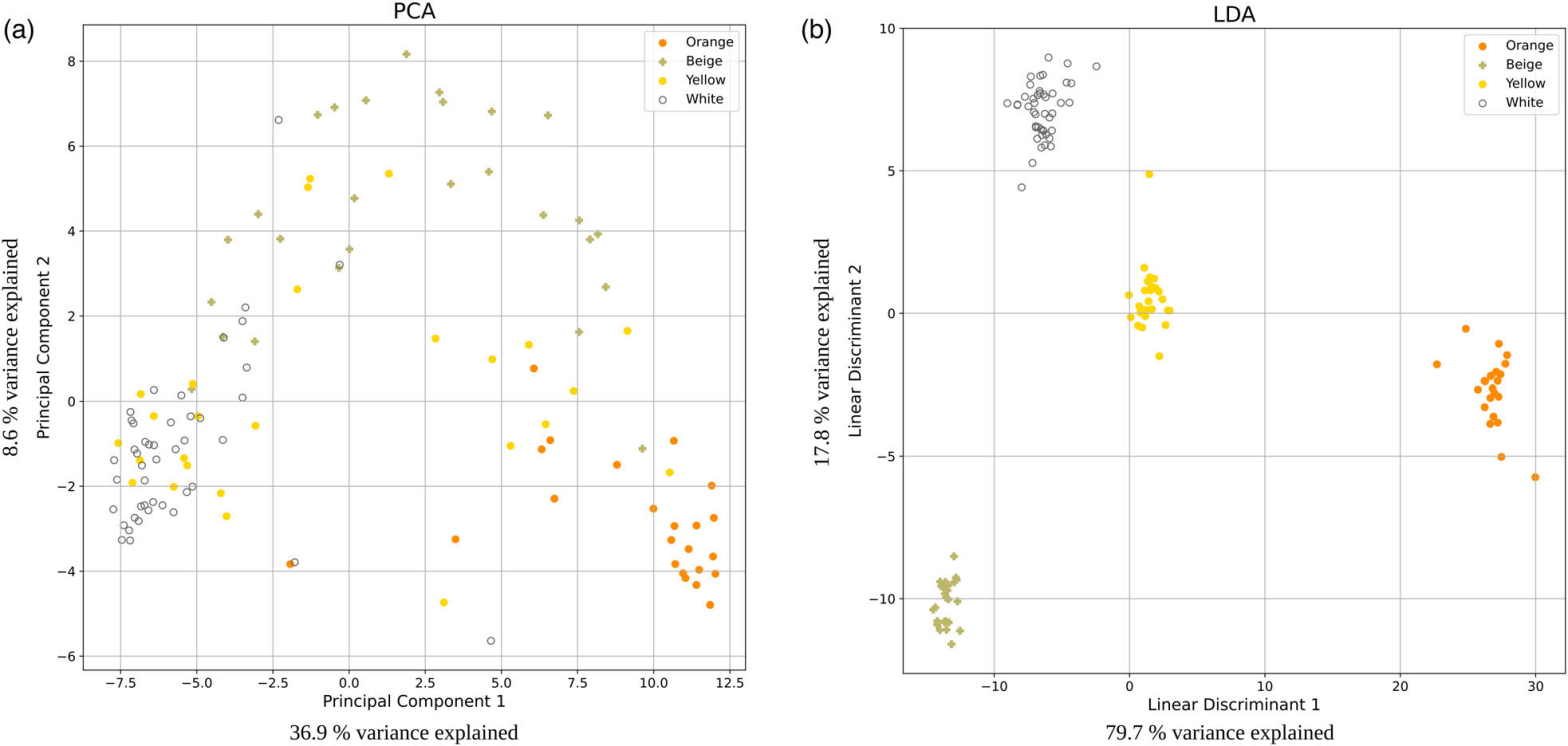
(a) Principal component analysis (PCA) and (b) linear discriminant analysis (LDA) of 129 SNP positions that increased the quality of at least nine independent XGBoost models using accessions with beige, orange, white and yellow seeds.

Once we had shown that a total of 129 SNPs separated quinoa accessions by seed colours for the three colours on which the machine learning model was built, we included the yellow‐seeded accessions and recalculated the dimensionality reductions. Interestingly, in the LDA the identified SNP set also clearly separated the yellow‐seeded accessions from the other three groups despite the yellow‐seed colour profile being far less homogenous compared to the other groups. In a PCA the yellow‐seeded accessions did not cluster together (Figure 3).

**Figure 4:**
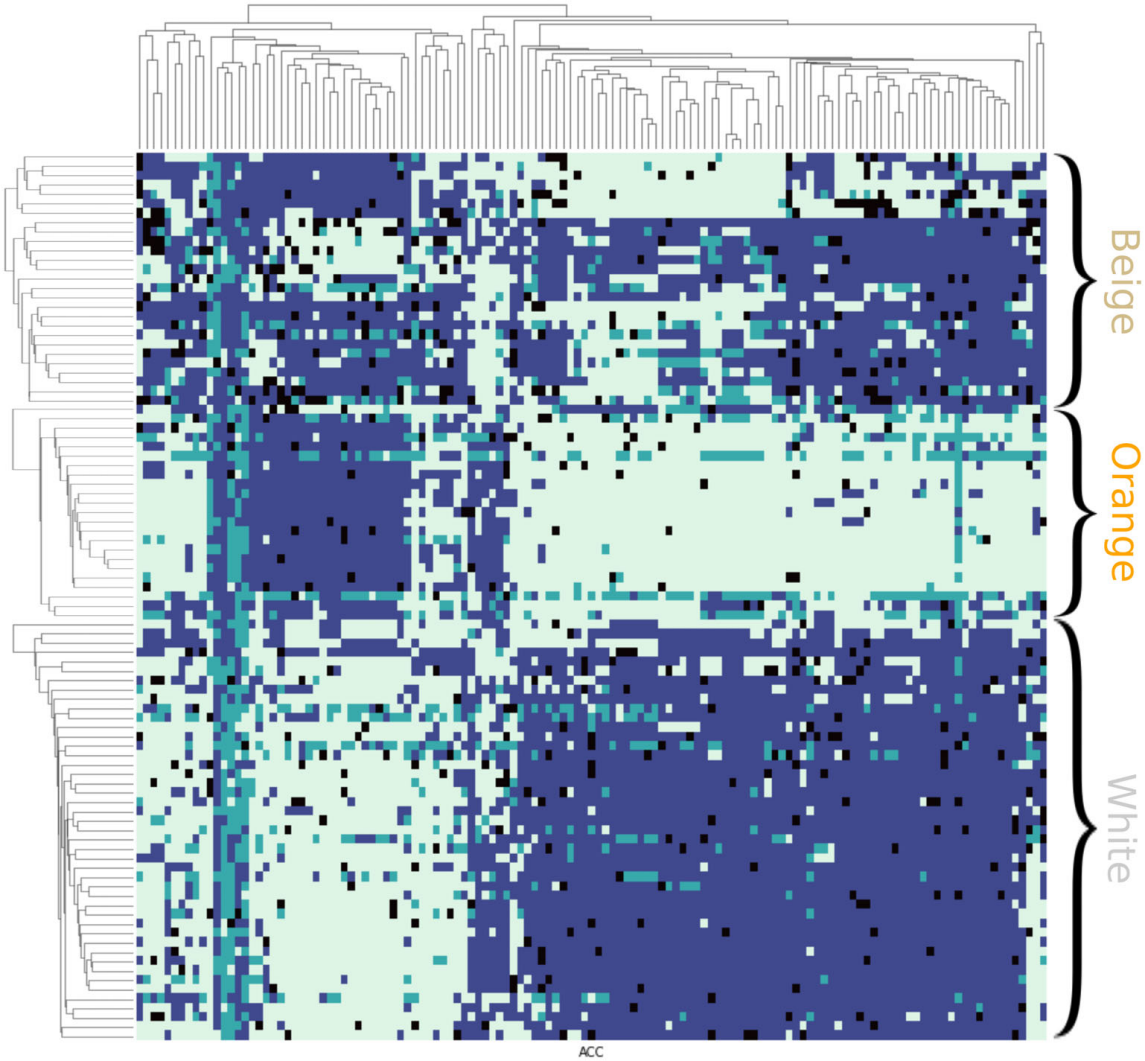
Hierarchical clustering of the genotypes at 129 variant positions discriminating beige‐, orange‐ and white‐seeded accessions. Dark blue: homozygous alternative; turquoise: heterozygous; light green: homozygous reference; black: missing data.

We compared the genotypes at the 129 variant positions between the three groups beige, orange and white (Figure 4; Table S2). The patterns that separated orange, white and beige accessions were clearly visible, however, the patterns also showed that seed colour in quinoa cannot be reduced to single variant positions but rather to a complex combination of variants.

**Figure 5:**
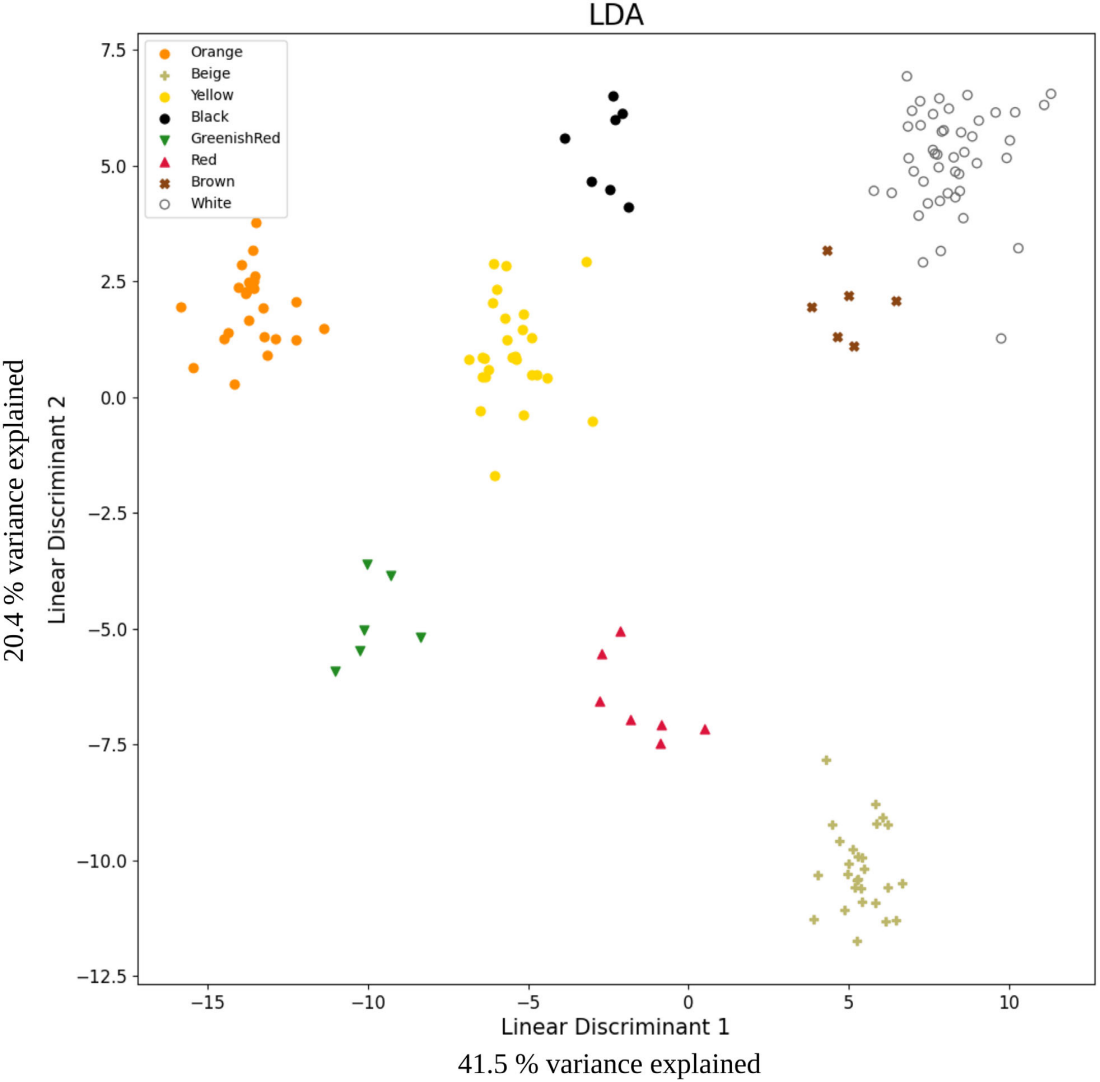
Linear discriminant analysis (LDA) of 129 SNP positions that increased the quality of at least nine independent XGBoost models. In addition to the four larger seed groups (beige, orange, white and yellow) we also included data from quinoa accessions with red, black, brown and greenish‐red seeds.

Next, we included the remaining small groups of seed colours black, red, brown and greenish‐red (six or seven accessions per group) in the LDA. The resulting clustering clearly separated all eight colour groups from each other solely based on the previously determined set of 129 variants (Figure 5). When including accessions that had a mixed seed colour profile, they could not be assigned to distinct groups (Figure S3).

### Comparison to univariate genome‐wide association (GWAS)

We compared these results to a classical univariate GWAS using linear mixed models (LMMs) as implemented in GEMMA (Zhou and Stephens, 2012). Since, to the best of our knowledge, all common univariate LMM algorithms do not allow multiclass categorical data analysis of non‐ordinal traits (typically, LMMs are directed towards binary classification problems or quantitative analyses) we conducted multiple rounds of GWAS by performing pairwise comparisons among all possible combinations of the three primary seed colours beige, orange and white. Subsequently, we applied stringent Bonferroni correction for multiple tests, yet even after correction, GWAS identified 1073 significant variants (6.8 times more than XGBoost; Table S3). Furthermore, we categorized the samples into meaningful clusters, such as variants segregating white‐seeded accessions from the others, distinguishing dark‐seeded accessions from those with bright seeds, and various other groupings, in order to encompass a diverse range of binary colour combinations for classical GWAS. However, these models did not yield PVE (Proportion of Variance Explained) estimates of sufficient utility to be considered useful (Table S4).

We also used XGBoost to train new models based on the 1073 SNPs predicted to be significant by the LMM resulting in a mean prediction accuracy of 76% among 100 models of which the best model achieved a prediction accuracy of 92%. The 1073 variants detected with the univariate GWAS were linked to 240 genes (Table S3), seven of which were the same as those identified using the gradient boosting algorithm (see below).

Principal Component Analysis (PCA) of the variants identified as significant by LMM additionally substantiated that the variants detected by the extreme gradient‐boosted models attained a visibly enhanced delineation of the three subgroups (Figure S4).

Our findings indicate that the 129 SNPs identified by the extreme gradient boosting algorithm exhibit the highest predictive power for seed colour in quinoa according to the methods applied. Consequently, we pursued our search for genes governing seed colour in quinoa utilizing this set of genetic variants.

### Candidate genes involved in seed pigmentation

The genomic location of the 129 SNPs that successfully separated the accessions by seed colour is of special interest to identify candidate genes involved in seed pigmentation. We determined collinear SNPs (correlation ≥97%) in the total set of 3.9 million variants and included the resulting 29 SNPs (collinear to nine out of the 129 predictive SNPs) in the analysis (Table S5). Of the total of 158 SNPs, we found 54 SNPs located in 41 genes or their flanking regions of 1000 bp (Table S6). The initial 129 SNPs were located in 40 genes or their flanking regions.

In total, 21 of the 41 genes were annotated with functions and GO terms, with 40% of them directly related to reproduction or seed development (Table S6). We used publicly available shotgun proteomics (Poza‐Viejo *et al*., 2023) and transcriptomics (Grimberg *et al*., 2022) data from quinoa seeds to assess the expression of the 41 candidate genes. Our results showed that 27 of the 41 candidates could be detected in the proteome and 39 of the 41 candidate genes could be detected in the transcriptome. Reads per kilobase of exon model per million mapped reads (RPKM) values were calculated for the selected candidate genes from the RNA‐seq data set related to the selected candidate genes. Notable disparities in RPKM values were observed among a yellow‐seeded, a red‐seeded and an orange‐seeded accession. These disparities suggest contrasting regulatory mechanisms for at least a subset of the candidate genes in our study (Table S6).

We compared our candidate genes against the seven known genes in the betalain synthesis pathway, as depicted in the KEGG database (Kanehisa, 2023), and found that variants within two of these were of high feature importance for seed colour differentiation by our models. One of them is the DODA gene encoding for a DOPA 4,5 dioxygenase that facilitates the formation of betalamic acid (the precursor for both betacyanin and betaxanthin) from L‐3,4‐dihydroxyphenylalanine (L‐DOPA). The second is catechol O‐methyltransferase (EC 2.1.1.6) which catalyses the formation of dopamine‐based betaxanthins (Figure S5). We found both transcripts and proteins expressed within seed tissues according to transcriptomics and proteomics data. No other genes within the betalain synthesis pathway contained variants that were informative for separation by seed colour. We also checked whether genes that play a role in the anthocyanin or tannin pathways contained variants informative for separating seeds by colour but no such variants were identified.

The SNP considered important for separating accessions by seed colour by the most independent models (38 out of 100) was in proximity to a gene located at position 531 270 of contig tig00008894 on quinoa chromosome CquChr18 and was flanked by the genes g47234 and g47235. We examined the functional annotation of these genes, which revealed that both belong to the cytochrome P450 gene family. The assigned GO terms for these two genes clearly linked them to a process involved in seed development, including the terms ‘development process involved in reproduction’, ‘fruit ripening’ and ‘developmental maturation’. Upon further inspection of the region, we found that these genes are part of a gene cluster comprising five consecutive cytochrome P450 genes (g47232‐g47236). Previous studies have shown that cytochrome P450 genes are often organized in large clusters of up to 15 genes and that these clusters play an important role in plant biosynthetic pathways (Werck‐Reichhart and Feyereisen, 2000). The genes g47233, g47234 and g47235 have pairwise protein sequence identities ranging from 73% to 85%. Within the genome of the Bolivian reference accession, both g47232 and g47236 appear to have been pseudogenised due to the presence of premature stop codons. We conducted a comparative analysis in the genome assembly of the Chilean quinoa accession QQ74 (Jarvis *et al*., 2017), where all five genes were found to be fully functional.

It has been described that CYP76AD1, a member of the cytochrome P450 family, controls the formation of betacyanin in the roots of table beet (Hatlestad *et al*., 2012) and in the stems of quinoa (Imamura *et al*., 2018). We analysed these genes for similarity and found that no gene within the identified cytochrome P450 gene cluster on CquChr18 is an ortholog of CYP76AD1. Along this line, we could not detect quinoa CYP76AD1 expression in recently published shotgun proteomics data from quinoa seeds (Poza‐Viejo *et al*., 2023). Thus, our data link seed colour to a novel cytochrome P450 gene cluster that is unrelated to CYP76AD1.

To better understand the roles of the genes within this cluster we also considered SNPs present in only three or more independent models and that were located within genes of the cytochrome gene cluster. This identified one variant that has large impact on protein sequence. This mutation was contained within the cytochrome P450 gene g47234 and was annotated as disrupting RNA splicing due to an A>T transversion in a splice donor site.

We then tested to what extent the five variants associated with the betalain biosynthetic pathway and the newly discovered cytochrome P450 genes are predictive for seed colour in quinoa. When recalculating all 100 models with these SNPs we observed a slight enhancement in AUC ROC from 0.82 to 0.85 and an increase in the mean prediction accuracy across 100 models from 71% to 73% as compared to the original input data of 3.9 million SNPs. However, each individual SNP of the total of 129 SNPs contributed to enhancing the prediction accuracy, so that when combined, the 129 SNPs yielded the highest prediction accuracy of 82%.

Notably, the two most important loci, that is, the locus encompassing DOPA 4,5‐dioxygenase (DODA) and the cytochrome P450 gene cluster, were among the six loci detected by both the univariate GWAS and the XGBoost approach, reinforcing their crucial role in controlling seed colour in quinoa.

## Discussion

In this work we used the power of gradient‐boosted machine learning models applied on complex plant whole‐genome sequencing data to elucidate the molecular basis of seed colour in quinoa.

Our models were trained on a phenotypic classification generated by visual inspections of 20–50 seeds per accession. We were aware that the accuracy of our visual inspection of seed colour is limited as compared to, for example, photometry, however, we aimed for locating genomic regions involved in controlling colour rather than for exact classification of single seeds. The resulting clusters were distinct enough to establish genotype to phenotype relationships.

We were able to calculate models that can accurately classify the seed colour of quinoa based on SNP patterns. Extensive hyperparameter tuning was performed and our final classification accuracy that separated white, beige and orange‐seeded quinoa accessions was 88%. We extracted all variants that improved the model in the training set and could thereby massively narrow down the number of candidate variants. We also tested if population structure had a confounding effect by adding the top 10 principal components (reflecting population structure) as ‘features’ to the input data so that they would appear of high ‘feature importance’ if population structure was predictive. In our data population structure had no obvious effect.

To avoid biases that might emerge through the choice of training and test data, respectively, we calculated 100 models with different training and test sets and considered only variants that were considered as important in at least nine of them. This led us to a final set of 129 variants that were highly predictive of seed colour. When we retrained our models with a feature space reduced to those variants, the performance of our models increased. The augmentation in prediction accuracy can likely be attributed to a reduction in overfitting, resulting from the constrained parameter space when using a limited set of input features. This robustly underscores the efficacy of the selected variants from our initial models in predicting seed colour in quinoa. While SNPs directly linked to genes in the established biosynthetic pathway exhibited a reasonably good prediction accuracy, we posit that the determination of colour is influenced by a broader array of genes within the quinoa genome. This hypothesis is substantiated by the notably superior prediction accuracy achieved when utilizing the set of 129 variants.

To confirm that our chosen variant set plays an important role in separating the quinoa groups with different seed colours we used PCA and LDA. Our results showed that the identified SNPs led to a perfect separation of the three large and cohesive seed colour groups (beige‐, white‐ and orange‐seeded accessions). When adding the yellow‐seeded quinoa plants we observed their grouping between the white and orange‐seeded plants in the PCA. For the LDA there was still a perfect separation of all four groups, with yellow‐seeded plants clustering towards the white ones. Notably, we could also obtain separate clusters of additional seed colour groups that were too small to be included during model generation, that is, red, brown, black and greenish‐red‐seeded accessions, which formed their own sub‐clusters in the final LDA. Taken together, this suggests that the set of 129 variants identified universally impacts seed colour in quinoa, regardless of the exact colouring in a specific subgroup.

We used the genomic location of the important SNPs to identify candidate genes. Besides two genes that are known to play a critical role in the betalain synthesis pathway, our method identified a cytochrome P450 gene cluster. This is especially intriguing since genes of the same family have been associated with the red colour in table beets and stem colour of quinoa. Our results suggest that different members of this gene family may facilitate cyclo‐DOPA synthesis within the betalain pathway in different tissues. Furthermore, a wide array of genes seems to be associated with this trait. Possibly, the tetraploid nature of the quinoa genome has led to this diversification of seed colours by altering the capacity of the betalain synthesis pathway within the two subgenomes. The genes that we identified represent interesting targets for downstream functional studies to determine whether they contribute to seed colour variation as well as targets for breeding programmes. Regardless of functional validation, the SNP set identified is highly predictive of seed colour and can therefore immediately be deployed for selective breeding. It is worth noting that our method is unlikely to be influenced by the tetraploid nature of the quinoa genome. As previously shown, short read mapping permits clear separation of the quinoa subgenomes (Jarvis *et al*., 2017; Schiavinato *et al*., 2021), and quinoa essentially behaves in mapping assays as a large diploid genome.

In summary, we provide important insight into the genetics of the agriculturally important quinoa trait seed colour. Our results show that it is possible to classify plants based on their seed colours using variants called from whole‐genome sequencing data and we outline a workflow that could be usable to analyse a wide variety of phenotypic traits, since gradient boosting is not limited towards categorical traits, but is also easily applicable to essential quantitative plant traits such as yield or biomass. When compared to classical univariate GWAS, extreme gradient boosting offers the advantage of simultaneously analysing multiclass categorical data in a single step and providing greater precision in detecting predictive variants. It also offers the advantage of eliminating the requirement for strict Bonferroni correction. While gradient‐boosted models do not necessitate assumptions about hereditary components, it is noteworthy that linear models offer the advantage of easily incorporating population structure into the modelling process (Enoma *et al*., 2022; Grinberg *et al*., 2020). Our method could identify SNPs located in genes known to play a critical role in the betalain synthesis pathway. The identified candidate genes included a novel cytochrome P450 gene that may explain betalain biosynthesis in quinoa seeds in addition to further genes that could control this trait or generally play a role in the morphogenesis of quinoa seeds. Our findings indicate that the determination of seed colour in quinoa is likely governed by a multitude of genes, as no single genetic variant or gene demonstrated predictive capability for seed colour. Considering the many functionally relevant loci that our method discovered, demonstration of causality may involve substantial efforts. The feasibility of gene editing in quinoa has already been demonstrated (Xiao *et al*., 2022). Multi‐gene editing within a given cultivar may provide a possible avenue for testing associations.

In order to select for traits of interest within breeding programmes, variants of high predictive value could be included in selection models. In our case, these would be the five variants associated with known genes of the betalain synthesis pathway including cytochrome P450 that already achieved 73% prediction accuracy. However, it would also be possible to use the entire set of 129 variants, and by means of genomic selection, variants discovered by our approach could be used for concerted selection at multiple loci in parallel. The feasibility of such endeavours has already been demonstrated for several plant species (Krishnappa *et al*., 2021). Breeding efforts that target quinoa seed colour may spark substantial interest, considering that quinoa is regarded as a ‘superfood’ that exhibits an ever‐rising demand on global markets. Since betalains are also biotechnologically interesting pigments these results could also be targets for breeding efforts that do not focus on quinoa solely as food but as a product, for example, food colour production or even for betalain production through heterologous expression.

## Methods

### Plant material, DNA isolation and genotyping

The genebank of IPK Gatersleben (https://gbis.ipk‐gatersleben.de/) was queried for entries of *Chenopodium* sp. or *C. quinoa* with origin in Bolivia and Peru, respectively, resulting in 952 different accessions. Of these, 156 were selected maximizing diversity by including different sampling locations. If accessions from the same locality were included, we chose accessions with different seed colours according to their passport data. Deposition of all accessions in the IPK genebank took place before the Nagoya Protocol came into effect. Plants were grown under greenhouse conditions. DNA from leaf material was isolated using a streamlined CTAB protocol (Saghai‐Maroof *et al*., 1984), followed by one step of chloroform extraction. After precipitation, DNA was dissolved in 1× TE (Tris–EDTA) pH 8.0.

### Seed colour phenotyping

We had the seeds readily available and phenotyped the seed colours by visual inspection. Three of the authors classified seed samples from 156 accessions by their respective colour and consulted each other on the grouping in case of uncertainty.

### Genomic sequencing

Genome sequencing was performed with Illumina technology (Illumina, San Diego, CA). Two hundred nanograms of genomic DNA quantified using a Qubit fluorometer (Life Technologies, Foster City, CA) were sheared to a peak size of 580 bp with a Covaris M220 Focused Ultrasonicator (Covaris, Woburn, MA). Thereafter, genomic sequencing libraries were prepared using a TruSeq Nano LT library preparation kit (Illumina) using indexed adapters. The quality and quantity of the libraries were assessed on a Bioanalyzer 2100 instrument (Agilent, Santa Clara, CA) using a DNA 1000 chip. Eight libraries with different barcodes were pooled into one sequencing lane, aiming at fivefold genomic coverage for each sequenced accession. Sequencing was done on the Illumina HiSeq 2500 instrument using v4 sequencing chemistry and a 2 × 125 nt paired‐end sequencing recipe.

### Data processing

Quality filtering and sequencing read trimming was done using trimmomatic v0.35 (Bolger *et al*., 2014) with the following settings: LEADING: 28 TRAILING:28 SLIDINGWINDOW: 5:15 MINLEN:50. Genome mapping was performed using Bowtie2 v 2.4.1 (Langmead and Salzberg, 2012) with the ‘very‐sensitive’ preset and ‐X 1500 against the unmasked reference genome assembly RefCHEN125. To perform combined variant calling the gatk v4.2.3.0 (van der Auwera and O'Connor, 2020) variant calling pipeline was used comprising of following steps: (i) gatk HaplotypeCaller, (ii) gatk CombineGVCFs, (iii) gatk GenotypeGVCFs, (iv) gatk SelectVariants. Filtering criteria for the SNPs were MQ > 30, MLEAF >0.01, DP > 300 < 1500, FS < 60, MQRankSum >12.5, ReadPosRankSum >8, BaseQRankSum >12.5, restrict‐alleles‐to BIALLELIC, max‐nocall‐fraction 0.1. The genotype matrix was calculated from the final vcf files using vcftools v0.1.16 (Danecek *et al*., 2011) with the −012 flag. This resulted in a genotype matrix based on the variant calling results, where ‘0’ represents a position that is homozygous reference, ‘1’ represents a position that is heterozygous and ‘2’ represents a position that is homozygous alternative.

The RNA sequencing data was derived from a study on quinoa seed quality (Grimberg *et al*., 2022). We mapped the raw sequencing data against the quinoa reference RefCHEN125 using HISAT2 (Kim *et al*., 2019) with standard parameters and calculated RPKM values for all our candidate genes.

Hierarchical clustering for Figure 4 was done using the clustermap function of the Python library seaborn (Waskom, 2021).

### Repeat annotation

Repeats were annotated using RepeatMasker v4.1.2‐p1 (Smit *et al*., 2013) and RepeatModeler v2.0.3 (Flynn *et al*., 2020) using the TETools container v1.5. The full Dfam database v3.6 (Storer *et al*., 2021) and RepBase v20181026 (Bao *et al*., 2015) were used for repeat classification. RepeatModeler was run with the parameter ‘‐LTRStruct’ which enables the optional LTR structural finder. RepeatMasker was run with the rmblast engine.

### Gene prediction and functional annotation

Genes were predicted using AUGUSTUS v3.5.0 (Stanke *et al*., 2008) with hints generated from RNA‐seq data. Multiple RNA‐seq libraries (SRP077770, SRP404502 and SRP375030) and an IsoSeq library (SRR14414933) were aligned to the RefCHEN125 assembly (Bodrug‐Schepers *et al*., 2021) using HISAT v2.2.1 (Kim *et al*., 2019) with parameter ‘‐‐dta’. Intron hints were generated from the alignment using the bam2hint script from AUGUSTUS. Additionally, StringTie v2.2.1 (Shumate *et al*., 2022) was used to assemble transcripts from the alignment and with a custom python script these were converted to exon hints. AUGUSTUS was run with species parameters optimized for Caryophyllales (Minoche *et al*., 2015), an extrinsic config file optimized for these types of hints and non‐default parameters ‘‐‐genemodel = complete’ and ‘‐‐noInFrameStop = true’. Functional annotation was done using eggNOG‐mapper v2.1.9 (Cantalapiedra *et al*., 2021) with DIAMOND v2.0.15.153 (Buchfink *et al*., 2021) and the eggNOG v5 database (Huerta‐Cepas *et al*., 2019). Genes supported by hints and genes supported by the functional annotation were combined to form the final gene annotation. Completeness was estimated using BUSCO v5.2.2 (Manni *et al*., 2021) in protein mode and the lineage datasets ‘eukaryota_odb10’ and ‘eudicots_odb10’. Comparison between gene sets was done using the output of blastp (Camacho *et al*., 2009) with output format 6 and added ‘qlen’ and ‘slen’ fields.

### Model selection

The complete data analysis pipeline was written in Python v3.7.6. To compute the models, the genotype matrix was separated into a training (plus validation) set comprising 71 accessions and a test set comprising 24 accessions using the scikit learn (Pedregosa *et al*., 2011) function train_test_split with stratified sampling and the random state 47. The validation set was used to perform hyperparameter optimization for the gradient boosting algorithm XGBoost using the Bayesian optimization algorithm hyperopt (Bergstra *et al*., 2015). In 250 trials the hyperparameters (max_depth, min_child_wheight, gamma, colsample_by_tree, subsample, learning_rate) were optimized. For each round, four‐fold cross‐validation using xgboost.cv was performed and the mean AUC ROC was computed. It is worth noting that hyperopt, being a Bayesian process, generally lacks perfect reproducibility. Our models exhibited stability in terms of prediction accuracy and f1 score after a minimum of 250 rounds of parameter optimization. Each model underwent individual parameter optimization to guarantee that there is no overlap between the testing set and the training/validation set, thus mitigating any potential bias in prediction accuracies. This computational‐intensive process is essential to maintain fairness in the reported accuracy results. All models were calculated with balanced weights to account for the unbalanced dataset and with early stopping (35 rounds). The model with the best AUC was selected. The final model accuracy was calculated by predicting the test set with the chosen model. Features were selected using the XGBoost get_booster().get_score(importance_type = ‘gain’) function. Collinear features were detected using pearsonr from the python library scipy (Virtanen *et al*., 2020). To test for possible effects of population structure we integrated the top 10 principal components as additional features to the input data set (Farooq *et al*., 2022; Zhao *et al*., 2012).

### Univariate GWAS

The univariate GWAS was calculated using LMMs as implemented in GEMMA (Zhou and Stephens, 2012). This was done in a pairwise fashion combining all binary combination of the seeds coloured beige, orange and white. For each LMM a centered relatedness matrix was calculated to correct for population structure (−gk 1). The LMMs were calculated using the Wald test as a frequentist (−lmm 1). Each model was then corrected for multiple testing using Bonferroni correction (*P* < 1 × 10^−8^).

### Principal component analysis (PCA) and linear discriminant analysis (LDA)

PCA and LDA were calculated both on the complete SNP set and on the selected features. Scikit learn functions PCA and LDA were used with their standard settings and n_components = 2.

### Computing resources

All models were calculated on a Linux computing cluster featuring a CentOS 6.7 operating system. Most calculations were performed on a computing node equipped with 56 cores (112 threads, 4 GHz) and 192 GB memory. Data preprocessing, mapping and variant calling were performed on the Vienna Scientific Cluster using multiple nodes equipped with 16 cores (32 threads) and 128 GB memory. Scripts to automate analysis steps were coded with bash v5.0. All models were written in Python v3.7.6.

## Conflicts of interest

None declared.

## Supporting information


**Figure S1** Principal component analysis (PCA) and linear discriminant analysis (LDA) of all 3.95 million SNP positions in quinoa accessions with beige, orange and white seeds.

**Figure S2** Principal component analysis (PCA) and linear discriminant analysis (LDA) of 123 SNP positions that increased the quality of a singular XGBoost model using quinoa accessions with beige, orange and white seeds.

**Figure S3** Linear discriminant analysis (LDA) including all quinoa accessions analysed in this study based on 129 SNP positions that increased the quality of at least nine independent XGBoost models in the classification of beige‐, orange‐ and white‐seeded accessions.

**Figure S4** Principal component analysis (PCA) of 1073 SNP positions identified as significant by an LMM‐based GWAS using quinoa accessions with beige, orange and white seeds.

**Figure S5** Betalain biosynthesis pathway as shown in the KEGG database (Kanehisa, 2023).

**Table S1** List of analysed quinoa accessions including information about their seed colour, sequencing coverage and number of homozygous (#hom) and heterozygous (#het) variant positions

**Table S2** List of 129 SNPs in the quinoa reference assembly RefCHEN125 that increased the quality of at least nine independent XGBoost models

**Table S3** Catalogue of significant variants to distinguish seed colour as detected by a univariate GWAS

**Table S4** Evaluation of predictive accuracies for the three distinct seed colours beige, orange and white

**Table S5** Variants linked to the 129 predictive variants with a Pearson correlation coefficient larger than or equal to 0.97

**Table S6** Catalogue of genes exhibiting one or more SNPs within the gene body or in close proximity (1000 bp flanking regions)

**Data S1** Supporting Information

## Data Availability

Python scripts for hyper‐parameter optimization, seed colour prediction, LDA and PCA are available on github: https://github.com/FLsandell/XGBquinoa. The genomic variation data for 156 quinoa accessions are available at figshare: https://doi.org/10.6084/m9.figshare.24466522.v1. Sequencing data generated in this study were deposited in the NCBI SRA database under BioProject PRJNA985441 with accession numbers SAMN35797054‐SAMN35797159. Source data are provided in this paper. The gene set and the functional annotation for RefCHEN125 are available at http://bioinformatics.boku.ac.at/.

## References

[pbi14267-bib-0001] van der Auwera, G. and O'Connor, B.D. (2020) Genomics in the Cloud: Using Docker, GATK, and WDL in Terra, 1st edn. Sebastopol, CA: O'Reilly Media.

[pbi14267-bib-0002] Bao, W. , Kojima, K.K. and Kohany, O. (2015) Repbase update, a database of repetitive elements in eukaryotic genomes. Mobile DNA, 6, 11. 26045719 10.1186/s13100-015-0041-9 PMC4455052

[pbi14267-bib-0004] Bergstra, J. , Komer, B. , Eliasmith, C. , Yamins, D. and Cox, D.D. (2015) Hyperopt: a Python library for model selection and hyperparameter optimization. Comput. Sci. Discov. 8, 014008.

[pbi14267-bib-0005] Bodrug‐Schepers, A. , Stralis‐Pavese, N. , Buerstmayr, H. , Dohm, J.C. and Himmelbauer, H. (2021) Quinoa genome assembly employing genomic variation for guided scaffolding. Theor. Appl. Genet. 134, 3577–3594. 34365519 10.1007/s00122-021-03915-x PMC8519820

[pbi14267-bib-0006] Bolger, A.M. , Lohse, M. and Usadel, B. (2014) Trimmomatic: a flexible trimmer for Illumina sequence data. Bioinformatics, 30, 2114–2120. 24695404 10.1093/bioinformatics/btu170 PMC4103590

[pbi14267-bib-0007] Brockington, S.F. , Yang, Y. , Gandia‐Herrero, F. , Covshoff, S. , Hibberd, J.M. , Sage, R.F. , Wong, G.K.S. *et al*. (2015) Lineage‐specific gene radiations underlie the evolution of novel betalain pigmentation in Caryophyllales. New Phytol. 207, 1170–1180. 25966996 10.1111/nph.13441 PMC4557044

[pbi14267-bib-0008] Buchfink, B. , Reuter, K. and Drost, H.‐G. (2021) Sensitive protein alignments at tree‐of‐life scale using DIAMOND. Nat. Methods, 18, 366–368. 33828273 10.1038/s41592-021-01101-x PMC8026399

[pbi14267-bib-0009] Calva‐Estrada, S.J. , Jiménez‐Fernández, M. and Lugo‐Cervantes, E. (2022) Betalains and their applications in food: the current state of processing, stability and future opportunities in the industry. Food Chem. Mol. Sci. 4, 100089. 10.1016/j.fochms.2022.100089 PMC8991513 35415668

[pbi14267-bib-0010] Camacho, C. , Coulouris, G. , Avagyan, V. , Ma, N. , Papadopoulos, J. , Bealer, K. and Madden, T.L. (2009) BLAST+: architecture and applications. BMC Bioinform. 10, 421. 10.1186/1471-2105-10-421 PMC2803857 20003500

[pbi14267-bib-0011] Cantalapiedra, C.P. , Hernández‐Plaza, A. , Letunic, I. , Bork, P. and Huerta‐Cepas, J. (2021) eggNOG‐mapper v2: functional annotation, orthology assignments, and domain prediction at the Metagenomic Scale. Mol. Biol. Evol. 38, 5825–5829. 34597405 10.1093/molbev/msab293 PMC8662613

[pbi14267-bib-0012] Chen, T. and Guestrin, C. (2016) XGBoost: A Scalable Tree Boosting System. *Proceedings of the 22nd ACM SIGKDD International Conference on Knowledge Discovery and Data Mining*, 785–794 10.1145/2939672.2939785

[pbi14267-bib-0013] Danecek, P. , Auton, A. , Abecasis, G. , Albers, C.A. , Banks, E. , DePristo, M.A. , Handsaker, R.E. *et al*. (2011) The variant call format and VCFtools. Bioinformatics, 27, 2156–2158. 21653522 10.1093/bioinformatics/btr330 PMC3137218

[pbi14267-bib-0014] Enoma, D.O. , Bishung, J. , Abiodun, T. , Ogunlana, O. and Osamor, V.C. (2022) Machine learning approaches to genome‐wide association studies. J. King Saud Univ. Sci. 34, 101847.

[pbi14267-bib-0015] Escribano, J. , Cabanes, J. , Jiménez‐Atiénzar, M. , Ibañez‐Tremolada, M. , Gómez‐Pando, L.R. , García‐Carmona, F. and Gandía‐Herrero, F. (2017) Characterization of betalains, saponins and antioxidant power in differently colored quinoa (*Chenopodium quinoa*) varieties. Food Chem. 234, 285–294. 28551238 10.1016/j.foodchem.2017.04.187

[pbi14267-bib-0016] Farooq, M. , Dijk, A.D.J.V. , Nijveen, H. , Mansoor, S. and De Ridder, D. (2022) Genomic prediction in plants: opportunities for machine learning‐based approaches. F1000Research, 18, 802. 10.12688/f1000research.122437.1 PMC10080209 37035464

[pbi14267-bib-0017] Fisher, R.A. (1936) The use of multiple measurements in taxonomic problems. Ann. Eugen. 7, 179–188.

[pbi14267-bib-0018] Flynn, J.M. , Hubley, R. , Goubert, C. , Rosen, J. , Clark, A.G. , Feschotte, C. and Smit, A.F. (2020) RepeatModeler2 for automated genomic discovery of transposable element families. Proc. Natl Acad. Sci. 117, 9451–9457. 32300014 10.1073/pnas.1921046117 PMC7196820

[pbi14267-bib-0019] Friedman, J.H. (2001) Greedy function approximation: a gradient boosting machine. Ann. Stat. 29, 1189–1232. 10.1214/aos/1013203451

[pbi14267-bib-0003] Gordillo‐Bastidas, E. , Díaz‐Rizzolo, D.A. , Roura, E. , Massanés, T. and Gomis, R. (2016) Quinoa (*Chenopodium quinoa* Willd), from nutritional value to potential health benefits: an integrative review. J. Nutr. Food Sci. 6, 1000497. 10.4172/2155-9600.1000497

[pbi14267-bib-0020] Grimberg, Å. , Saripella, G.V. , Repo‐Carrasco Valencia, R.A.‐M. , Bengtsson, T. , Alandia, G. and Carlsson, A.S. (2022) Transcriptional regulation of quinoa seed quality: identification of novel candidate genetic markers for increased protein content. Front. Plant Sci. 13, 816425. 35720573 10.3389/fpls.2022.816425 PMC9201758

[pbi14267-bib-0021] Grinberg, N.F. , Orhobor, O.I. and King, R.D. (2020) An evaluation of machine‐learning for predicting phenotype: studies in yeast, rice, and wheat. Mach. Learn. 109, 251–277. 32174648 10.1007/s10994-019-05848-5 PMC7048706

[pbi14267-bib-0022] Hatlestad, G.J. , Sunnadeniya, R.M. , Akhavan, N.A. , Gonzalez, A. , Goldman, I.L. , McGrath, J.M. and Lloyd, A.M. (2012) The beet *R* locus encodes a new cytochrome P450 required for red betalain production. Nat. Genet. 44, 816–820. 22660548 10.1038/ng.2297

[pbi14267-bib-0023] Huerta‐Cepas, J. , Szklarczyk, D. , Heller, D. , Hernández‐Plaza, A. , Forslund, S.K. , Cook, H. , Mende, D.R. *et al*. (2019) eggNOG 5.0: a hierarchical, functionally and phylogenetically annotated orthology resource based on 5090 organisms and 2502 viruses. Nucleic Acids Res. 47(D1), D309–D314. 30418610 10.1093/nar/gky1085 PMC6324079

[pbi14267-bib-0024] Imamura, T. , Takagi, H. , Miyazato, A. , Ohki, S. , Mizukoshi, H. and Mori, M. (2018) Isolation and characterization of the betalain biosynthesis gene involved in hypocotyl pigmentation of the allotetraploid *Chenopodium quinoa* . Biochem. Biophys. Res. Commun. 496, 280–286. 29317207 10.1016/j.bbrc.2018.01.041

[pbi14267-bib-0025] Jarvis, D.E. , Ho, Y.S. , Lightfoot, D.J. , Schmöckel, S.M. , Li, B. , Borm, T.J.A. , Ohyanagi, H. *et al*. (2017) The genome of *Chenopodium quinoa* . Nature, 542, 307–312. 28178233 10.1038/nature21370

[pbi14267-bib-0058] Kanehisa, M. , Furumichi, M. , Sato, Y. , Kawashima, M. and Ishiguro‐Watanabe, M. (2023) KEGG for taxonomy‐based analysis of pathways and genomes. Nucleic Acids Res. 51(D1), D587–D592. 10.1093/nar/gkac963 36300620 PMC9825424

[pbi14267-bib-0026] Kim, D. , Paggi, J.M. , Park, C. , Bennett, C. and Salzberg, S.L. (2019) Graph‐based genome alignment and genotyping with HISAT2 and HISAT‐genotype. Nat. Biotechnol. 37, 907–915. 31375807 10.1038/s41587-019-0201-4 PMC7605509

[pbi14267-bib-0027] Krishnappa, G. , Savadi, S. , Tyagi, B.S. , Singh, S.K. , Mamrutha, H.M. , Kumar, S. , Mishra, C.N. *et al*. (2021) Integrated genomic selection for rapid improvement of crops. Genomics, 113, 1070–1086. 33610797 10.1016/j.ygeno.2021.02.007

[pbi14267-bib-0028] Langmead, B. and Salzberg, S.L. (2012) Fast gapped‐read alignment with Bowtie 2. Nat. Methods, 9, 357–359. 22388286 10.1038/nmeth.1923 PMC3322381

[pbi14267-bib-0029] Lechner, J.F. and Stoner, G.D. (2019) Red beetroot and betalains as cancer chemopreventative agents. Molecules, 24, 1602. 31018549 10.3390/molecules24081602 PMC6515411

[pbi14267-bib-0030] Manni, M. , Berkeley, M.R. , Seppey, M. , Simão, F.A. and Zdobnov, E.M. (2021) BUSCO update: novel and streamlined workflows along with broader and deeper phylogenetic coverage for scoring of eukaryotic, prokaryotic, and viral genomes. Mol. Biol. Evol. 38, 4647–4654. 34320186 10.1093/molbev/msab199 PMC8476166

[pbi14267-bib-0031] Minoche, A.E. , Dohm, J.C. , Schneider, J. , Holtgräwe, D. , Viehöver, P. , Montfort, M. , Rosleff Sörensen, T. *et al*. (2015) Exploiting single‐molecule transcript sequencing for eukaryotic gene prediction. Genome Biol. 16, 184. 26328666 10.1186/s13059-015-0729-7 PMC4556409

[pbi14267-bib-0032] Novakovsky, G. , Dexter, N. , Libbrecht, M.W. , Wasserman, W.W. and Mostafavi, S. (2023) Obtaining genetics insights from deep learning via explainable artificial intelligence. Nat. Rev. Genet. 24, 125–137. 36192604 10.1038/s41576-022-00532-2

[pbi14267-bib-0033] Patiranage, D.S. , Rey, E. , Emrani, N. , Wellman, G. , Schmid, K. , Schmöckel, S.M. , Tester, M. *et al*. (2022) Genome‐wide association study in quinoa reveals selection pattern typical for crops with a short breeding history. Elife, 11, e66873. 35801689 10.7554/eLife.66873 PMC9388097

[pbi14267-bib-0034] Pearson, K. (1901) LIII. On lines and planes of closest fit to systems of points in space. London Edinburgh Dublin Philos. Magaz. J. Sci. 2, 559–572.

[pbi14267-bib-0035] Pedregosa, F. , Varoquaux, G. , Gramfort, A. , Michel, V. , Thirion, B. , Grisel, O. , Blondel, M. *et al*. (2011) Scikit‐learn: machine learning in Python. J. Mach. Learn. Res. 12, 2825–2830.

[pbi14267-bib-0036] Pickersgill, B. (2007) Domestication of plants in the Americas: insights from mendelian and molecular genetics. Ann. Bot. 100, 925–940. 17766847 10.1093/aob/mcm193 PMC2759216

[pbi14267-bib-0037] Poza‐Viejo, L. , Redondo‐Nieto, M. , Matías, J. , Granado‐Rodríguez, S. , Maestro‐Gaitán, I. , Cruz, V. , Olmos, E. *et al*. (2023) Shotgun proteomics of quinoa seeds reveals chitinases enrichment under rainfed conditions. Sci. Rep. 13, 4951. 36973333 10.1038/s41598-023-32114-5 PMC10043034

[pbi14267-bib-0038] Quinoa production Bolivia 2020 . (n.d.) Statista. Retrieved November 17, 2022, from https://www.statista.com/statistics/520749/quinoa‐production‐in‐bolivia/

[pbi14267-bib-0039] Quinoa production in Peru 2020 . (n.d.) Statista. Retrieved November 17, 2022, from https://www.statista.com/statistics/518477/quinoa‐production‐peru/

[pbi14267-bib-0040] Saghai‐Maroof, M.A. , Soliman, K.M. , Jorgensen, R.A. and Allard, R.W. (1984) Ribosomal DNA spacer‐length polymorphisms in barley: Mendelian inheritance, chromosomal location, and population dynamics. Proc. Natl Acad. Sci. 81, 8014–8018. 6096873 10.1073/pnas.81.24.8014 PMC392284

[pbi14267-bib-0041] Schiavinato, M. , Bodrug‐Schepers, A. , Dohm, J.C. and Himmelbauer, H. (2021) Subgenome evolution in allotetraploid plants. Plant J. 106, 672–688. 33547826 10.1111/tpj.15190 PMC8251528

[pbi14267-bib-0042] Shen, X. , Jiang, C. , Wen, Y. , Li, C. and Lu, Q. (2022) A brief review on deep learning applications in genomic studies. Front. Syst. Biol. 2, 877717.

[pbi14267-bib-0043] Shumate, A. , Wong, B. , Pertea, G. and Pertea, M. (2022) Improved transcriptome assembly using a hybrid of long and short reads with StringTie. PLoS Comput. Biol. 18, e1009730. 35648784 10.1371/journal.pcbi.1009730 PMC9191730

[pbi14267-bib-0044] Simão, F.A. , Waterhouse, R.M. , Ioannidis, P. , Kriventseva, E.V. and Zdobnov, E.M. (2015) BUSCO: assessing genome assembly and annotation completeness with single‐copy orthologs. Bioinformatics, 31, 3210–3212. 26059717 10.1093/bioinformatics/btv351

[pbi14267-bib-0045] Smit, A. , Hubley, R. and Green, P. (2013) RepeatMasker Open‐4.0. http://www.repeatmasker.org

[pbi14267-bib-0046] Stafford, H.A. (1994) Anthocyanins and betalains: evolution of the mutually exclusive pathways. Plant Sci. 101, 91–98.

[pbi14267-bib-0047] Stanke, M. , Diekhans, M. , Baertsch, R. and Haussler, D. (2008) Using native and syntenically mapped cDNA alignments to improve *de novo* gene finding. Bioinformatics, 24, 637–644. 18218656 10.1093/bioinformatics/btn013

[pbi14267-bib-0048] Storer, J. , Hubley, R. , Rosen, J. , Wheeler, T.J. and Smit, A.F. (2021) The Dfam community resource of transposable element families, sequence models, and genome annotations. Mobile DNA, 12, 2. 33436076 10.1186/s13100-020-00230-y PMC7805219

[pbi14267-bib-0049] Timoneda, A. , Feng, T. , Sheehan, H. , Walker‐Hale, N. , Pucker, B. , Lopez‐Nieves, S. , Guo, R. *et al*. (2019) The evolution of betalain biosynthesis in Caryophyllales. New Phytol. 224, 71–85. 31172524 10.1111/nph.15980

[pbi14267-bib-0050] Virtanen, P. , Gommers, R. , Oliphant, T.E. , Haberland, M. , Reddy, T. , Cournapeau, D. , Burovski, E. *et al*. (2020) SciPy 1.0: fundamental algorithms for scientific computing in Python. Nat. Methods, 17, 261–272. 32015543 10.1038/s41592-019-0686-2 PMC7056644

[pbi14267-bib-0051] Waskom, M. (2021) seaborn: Statistical data visualization. J. Open Sour. Softw. 6, 3021.

[pbi14267-bib-0052] Werck‐Reichhart, D. and Feyereisen, R. (2000) Cytochromes P450: a success story. Genome Biol. 1, reviews3003.1. 11178272 10.1186/gb-2000-1-6-reviews3003 PMC138896

[pbi14267-bib-0053] Xiao, X. , Meng, F. , Satheesh, V. , Xi, Y. and Lei, M. (2022) An Agrobacterium‐mediated transient expression method contributes to functional analysis of a transcription factor and potential application of gene editing in *Chenopodium quinoa* . Plant Cell Rep. 41, 1975–1985. 35829752 10.1007/s00299-022-02902-w

[pbi14267-bib-0054] Yangquanwei, Z. , Neethirajan, S. and Karunakaran, C. (2013) Cytogenetic analysis of quinoa chromosomes using nanoscale imaging and spectroscopy techniques. Nanoscale Res. Lett. 8, 463. 24191931 10.1186/1556-276X-8-463 PMC4228249

[pbi14267-bib-0055] Zhao, Y. , Chen, F. , Zhai, R. , Lin, X. , Wang, Z. , Su, L. and Christiani, D.C. (2012) Correction for population stratification in random forest analysis. Int. J. Epidemiol. 41, 1798–1806. 23148107 10.1093/ije/dys183 PMC3535752

[pbi14267-bib-0056] Zhou, X. and Stephens, M. (2012) Genome‐wide efficient mixed‐model analysis for association studies. Nat. Genet. 44, 821–824. 22706312 10.1038/ng.2310 PMC3386377

[pbi14267-bib-0057] Zou, C. , Chen, A. , Xiao, L. , Muller, H.M. , Ache, P. , Haberer, G. , Zhang, M. *et al*. (2017) A high‐quality genome assembly of quinoa provides insights into the molecular basis of salt bladder‐based salinity tolerance and the exceptional nutritional value. Cell Res. 27, 1327–1340. 28994416 10.1038/cr.2017.124 PMC5674158

