# Supplementary figures and images for: Genomic basis of seed colour in quinoa inferred from variant patterns using extreme gradient boosting
Source: Plant Biotechnol J. 2024 Jan 11;22(5):1312–24. doi: 10.1111/pbi.14267 (PMC11022794; doi:10.1111/pbi.14267)

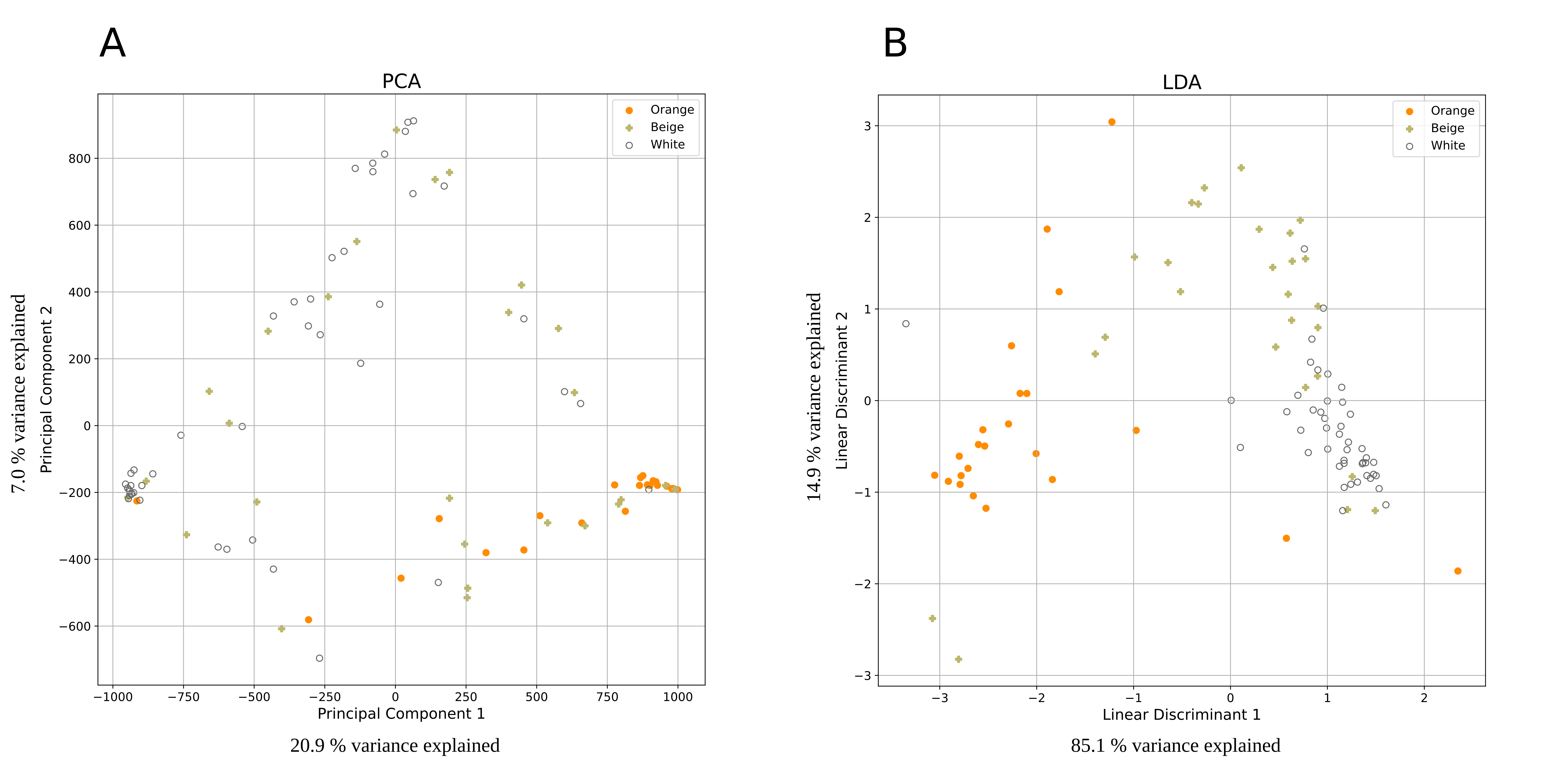

Supplement: Supplementary file 1 — Figure S1 Principal component analysis (PCA) and linear discriminant analysis (LDA) of all 3.95 million SNP positions in quinoa accessions with beige, orange and white seeds. [file PBI-22-1312-s009.png]

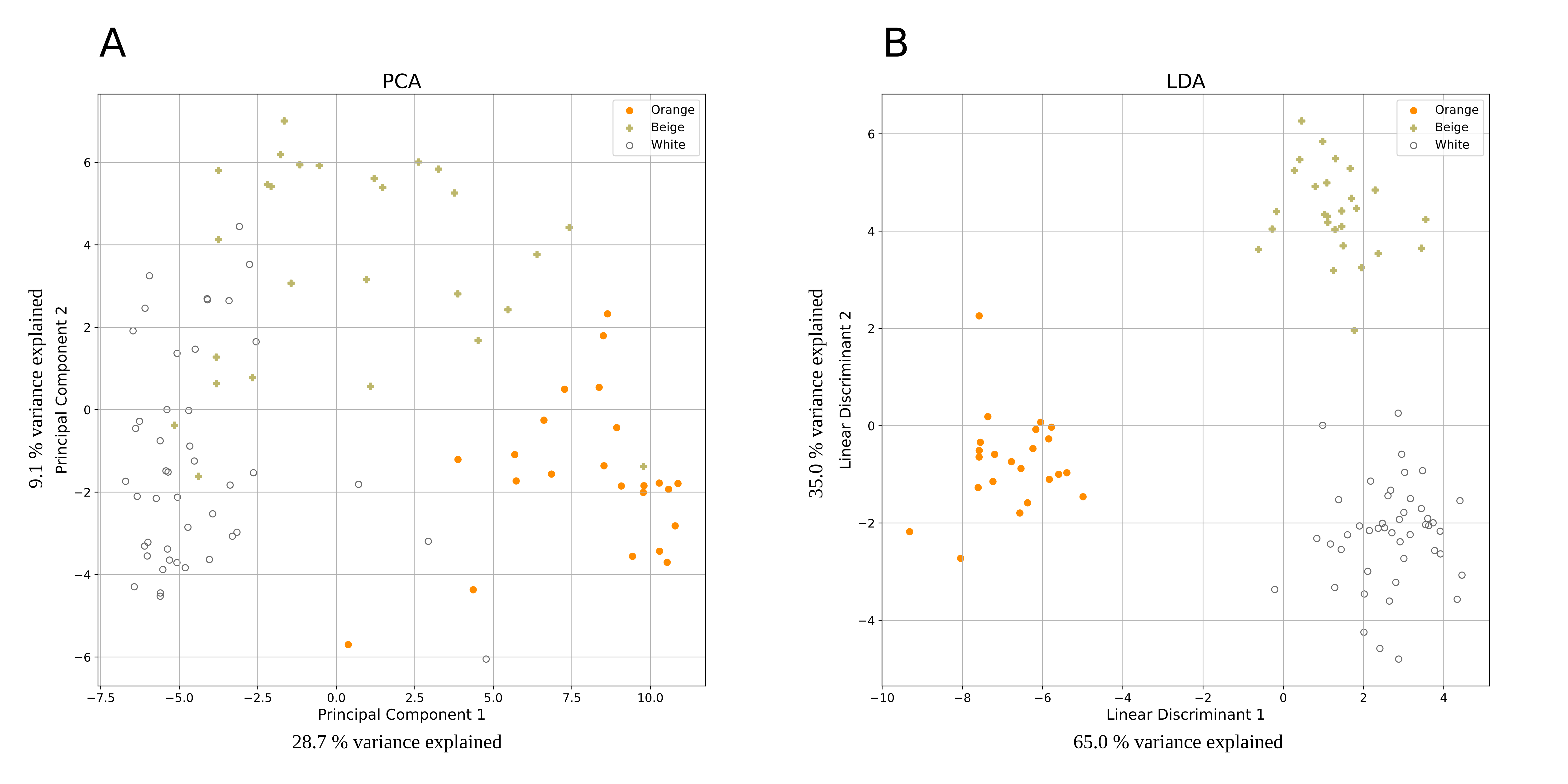

Supplement: Supplementary file 2 — Figure S2 Principal component analysis (PCA) and linear discriminant analysis (LDA) of 123 SNP positions that increased the quality of a singular XGBoost model using quinoa accessions with beige, orange and white seeds. [file PBI-22-1312-s012.png]

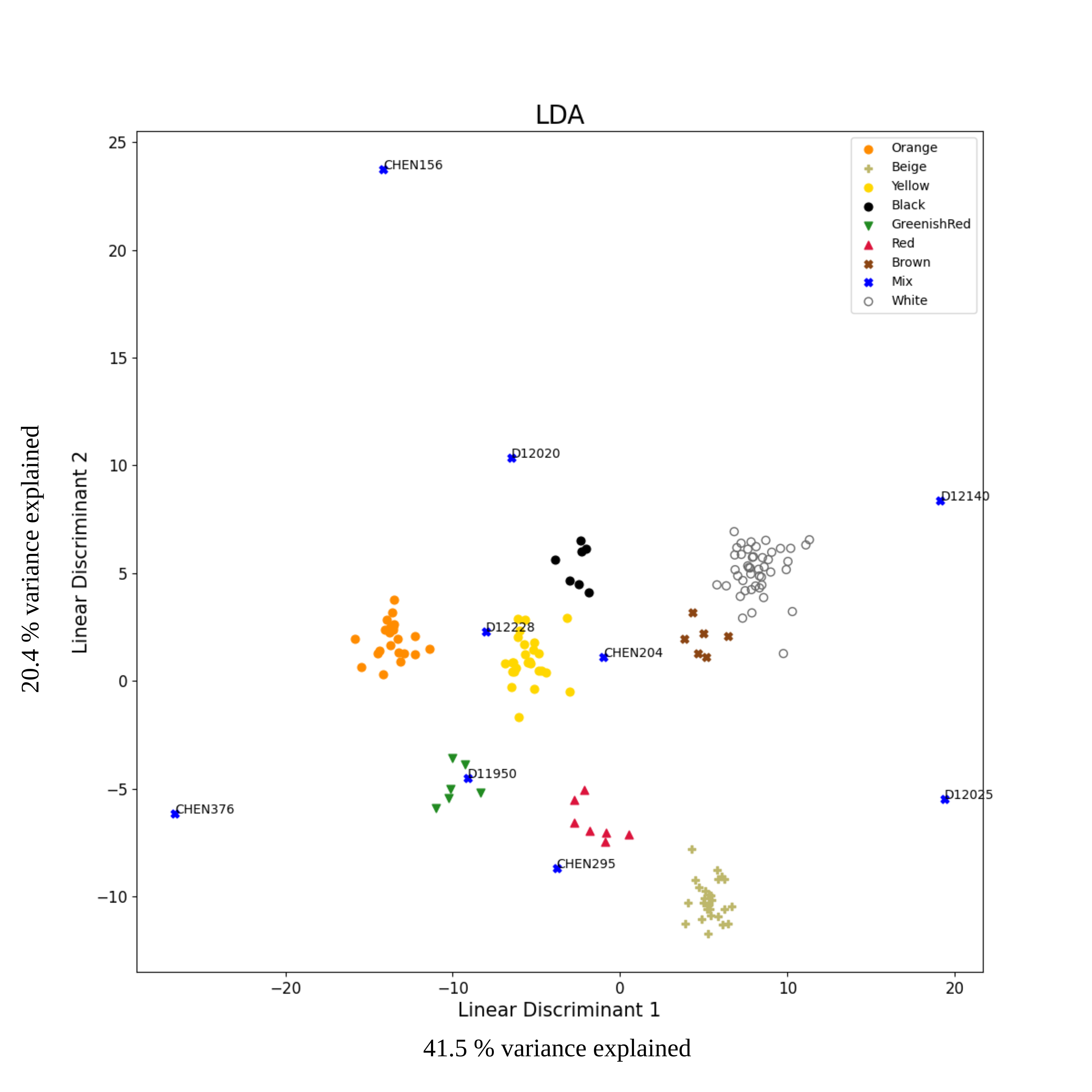

Supplement: Supplementary file 3 — Figure S3 Linear discriminant analysis (LDA) including all quinoa accessions analysed in this study based on 129 SNP positions that increased the quality of at least nine independent XGBoost models in the classification of beige‐, orange‐ and white‐seeded accessions. [file PBI-22-1312-s002.png]

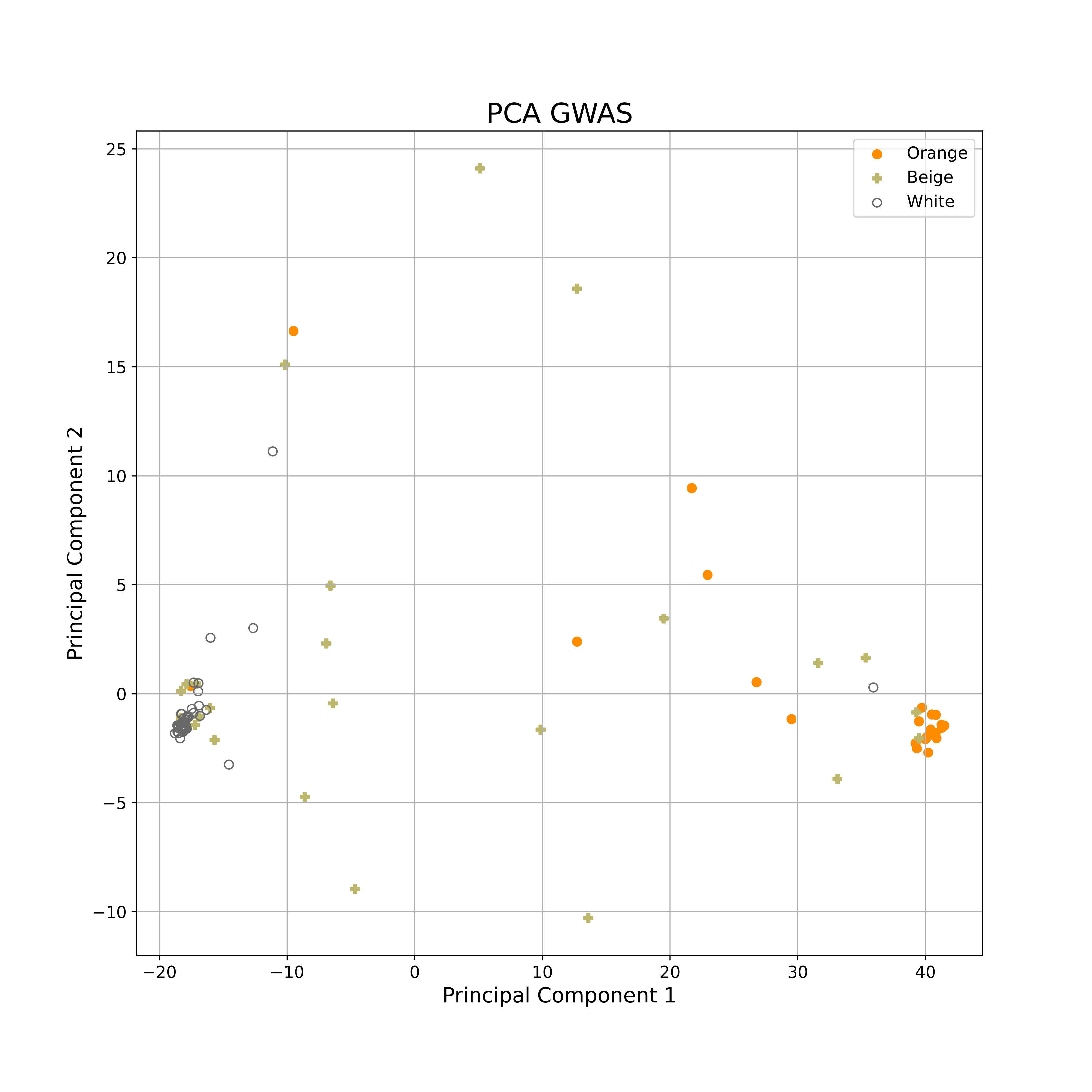

Supplement: Supplementary file 4 — Figure S4 Principal component analysis (PCA) of 1073 SNP positions identified as significant by an LMM‐based GWAS using quinoa accessions with beige, orange and white seeds. [file PBI-22-1312-s005.png]

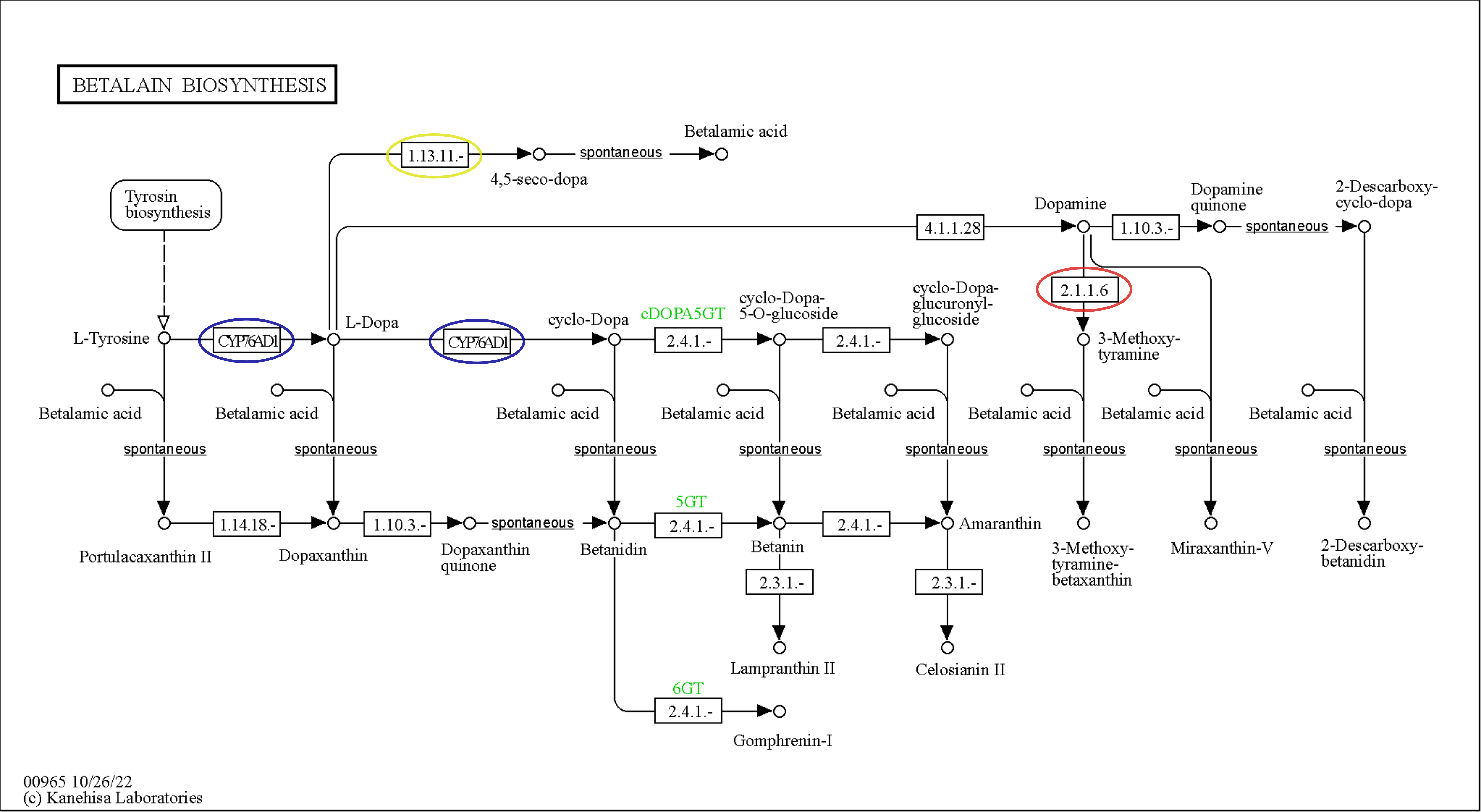

Supplement: Supplementary file 5 — Figure S5 Betalain biosynthesis pathway as shown in the KEGG database (Kanehisa, 2023). [file PBI-22-1312-s010.png]
